# Differential associations of novel metaflammation indices with incident stroke across traditional Chinese medicine constitutions: a retrospective community-based cohort study of older adults

**DOI:** 10.3389/fneur.2026.1750848

**Published:** 2026-06-03

**Authors:** Yang Li, Jun Ni, You Li, Suyan Zhao, Yun Chen

**Affiliations:** 1 Department of General Practice, Shanghai East Hospital, Tongji University School of Medicine, Shanghai, China; 2 Department of Geriatrics, Shanghai East Hospital, Tongji University School of Medicine, Shanghai, China; 3 Pudong New Area Doming Community Health Service Center, Shanghai, China

**Keywords:** cohort study, metaflammation, older adults, stroke, traditional Chinese medicine constitution

## Abstract

**Objective:**

To examine associations between novel metaflammation indices (LHR, MHR, NHR, PHR) and first stroke risk in community-dwelling older adults, and assess their differences across Traditional Chinese medicine (TCM) constitution types.

**Methods:**

A retrospective cohort study included 2,999 stroke-free adults (≥65 years) followed for 4 years. Data were collected through standardized questionnaires, physical examinations, and blood tests. Multivariable Cox proportional hazards models were used to evaluate the associations between these indices and incident stroke. Stratified analyses were conducted according to TCM constitution types.

**Results:**

During the follow-up period, 36 incident stroke cases were recorded. After multivariable adjustment, LHR (HR = 1.68, 95% CI: 1.09–2.61) and PHR (HR = 1.01, 95% CI: 1.00–1.01) were significantly positively associated with stroke risk in the overall population. Individuals with high-risk biased constitutions had a significantly elevated stroke risk (HR = 3.42, 95% CI: 1.32–8.80). Stratified analysis suggested that the associations of LHR and PHR with stroke were statistically significant in the balanced constitution and low-risk biased constitution subgroups, but not in the high-risk biased constitution subgroup. However, no significant interaction was observed.

**Conclusion:**

LHR and PHR may be associated with incident stroke in community-dwelling older adults, and their associations may vary across TCM constitution types. Therefore, integrating traditional risk factors, metaflammation indices, and TCM constitution identification might contribute to developing a more refined and personalized stroke risk assessment strategy. However, no significant interaction was observed, indicating that these variations should be interpreted cautiously and require further validation.

## 1 Introduction

Stroke is one of the leading causes of death and disability among older adults worldwide, posing severe challenges to socioeconomic and healthcare systems (1). Although significant progress has been made in the management of traditional cardiovascular risk factors such as hypertension, diabetes, and dyslipidemia (1, 2), these conventional risk factors alone are insufficient for comprehensively assessing stroke risk in the elderly population (3). This highlights the limitations of current risk prediction models and underscores the urgent need to develop novel biomarkers and more precise stratification strategies (4–6).

Metaflammation, a state of chronic, low-grade systemic inflammation, is a core pathological mechanism driving the pathogenesis of cardiovascular diseases (CVDs), including stroke (7). It originates from metabolic dysregulation (e.g., obesity, insulin resistance) and promotes vascular endothelial dysfunction, atherosclerosis, and plaque instability through the activation of innate immunity, release of pro-inflammatory cytokines (e.g., IL-6, TNF-α), and dysregulation of adipokines (8–14). In recent years, composite hematological indices that integrate pathways of inflammation (blood cell counts) and metabolic homeostasis (high-density lipoprotein cholesterol, HDL-C)—such as the Lymphocyte-to-HDL-C ratio (LHR), Monocyte-to-HDL-C ratio (MHR), Neutrophil-to-HDL-C ratio (NHR), and Platelet-to-HDL-C ratio (PHR)—have garnered significant attention in cardiovascular risk prediction due to their accessibility, cost-effectiveness, and potential as comprehensive biomarkers of systemic metaflammation (15–21). However, prospective evidence specifically linking these indices to incident stroke risk in community-dwelling elderly populations remains relatively scarce, and the consistency of their predictive performance across populations with varying inherent physiological states requires further in-depth investigation.

Traditional Chinese Medicine (TCM) constitution theory provides a holistic, individual-centered framework for evaluating inherent susceptibility to disease by integrating physiological, psychological, and socio-environmental characteristics (22, 23). According to the “Nine Body Constitution Theory” developed by Wang Qi and colleagues, TCM constitutions are categorized into one balanced type—the Gentleness constitution (GTC)—and eight biased types, including Qi-deficiency constitution (QDC), Yang-deficiency constitution (YaDC), Yin-deficiency constitution (YiDC), Phlegm-dampness constitution (PDC), Damp-heat constitution (DHC), Blood-stasis constitution (BSC), Qi-stagnation constitution (QSC), and Special-diathesis constitution (SDC) (24, 25). Substantial evidence links specific biased constitutions to endothelial dysfunction, insulin resistance, hypertension, diabetes mellitus, metabolic syndrome, sarcopenia, coronary heart disease (CHD) and metabolic dysfunction-associated fatty liver disease (MAFLD) (26–33). Nonetheless, whether the distinct pathophysiological profiles associated with different TCM constitutions confer differential associations between novel metaflammation indices and incident stroke risk remains unexplored. We hypothesize that the predictive utility of these indices is attenuated among individuals with high-risk biased constitutions (e.g., Yang-deficiency and Damp-heat), which may already be in a state of persistent, low-grade metaflammation and metabolic dysregulation, thereby limiting the additional predictive information these indices can provide. Conversely, stronger associations are expected in those with the Gentleness constitution (GTC) or lower-risk constitutions, where these indices might more sensitively capture the early transition toward a pro-inflammatory and metabolically disturbed state.

Accordingly, the present retrospective community-based cohort study was designed to assess the associations between novel metaflammation indices and incident stroke in older adults, and to examine whether these associations vary significantly across baseline TCM constitution types. By integrating modern metaflammation biomarkers with TCM constitution theory, this research aims to identify previously underappreciated sources of differences in stroke risk and contribute real-world evidence for targeted prevention strategies.

## 2 Methods

### 2.1 Study design and participants

**Figure 1:**
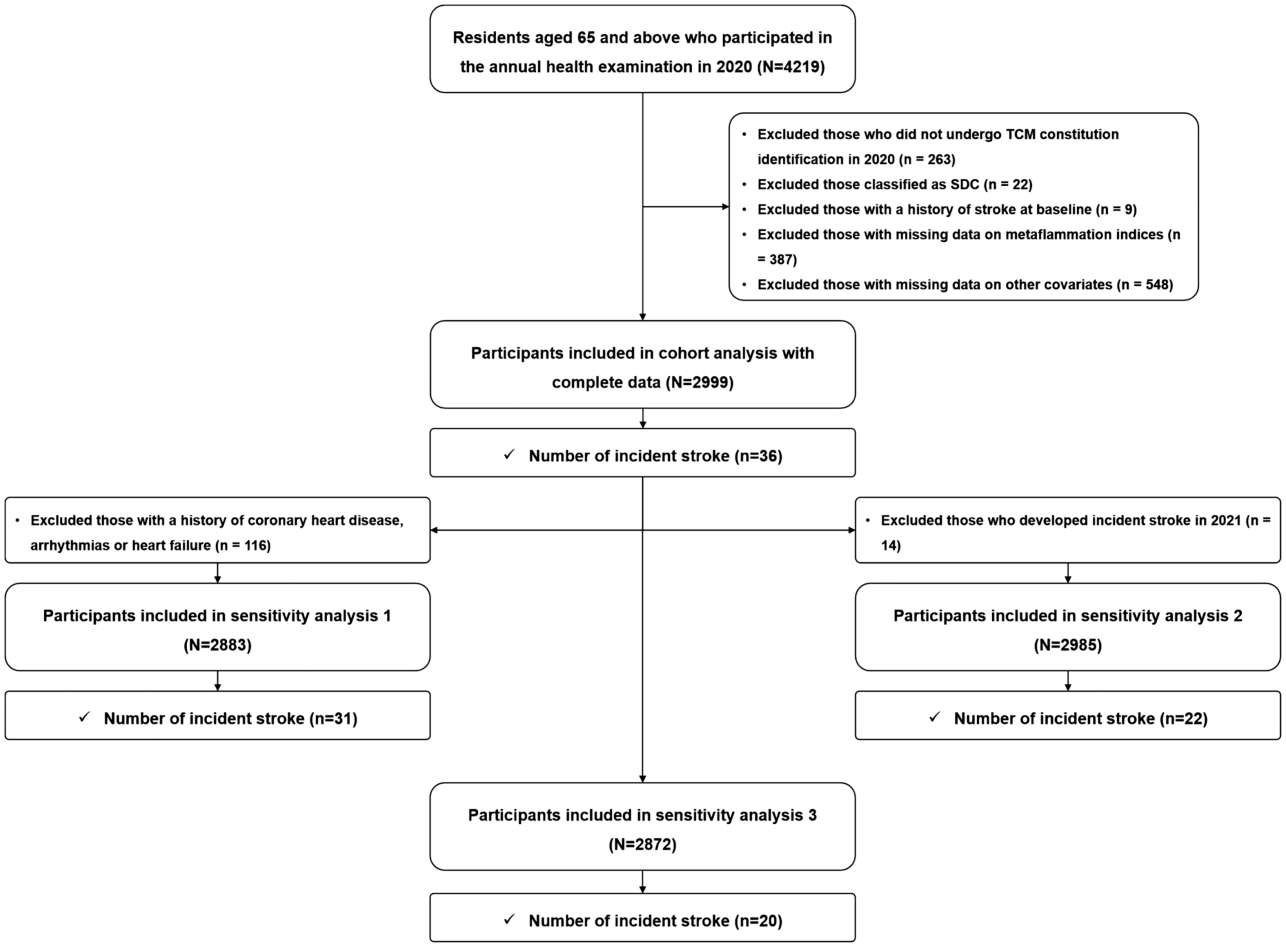
Flowchart of participants’ screening.

This retrospective cohort study utilized data from the annual health examination database of Dongming Community Health Service Center in Pudong New Area, Shanghai. The study initially included residents aged 65 and above who participated in the annual health examination in 2020 (*n* = 4,219). Major chronic disease diagnoses were identified for all participants based on electronic medical records from the community health service center. After excluding individuals who did not undergo TCM constitution identification in 2020 (*n* = 263), those classified as SDC (*n* = 22), those with a history of stroke at baseline (*n* = 9), those with missing data on metaflammation indices (*n* = 387), and those with missing data on other covariates (*n* = 548), a total of 2,999 participants were included in the cohort analysis. Follow-up was conducted annually through the end of each year by reviewing updated electronic medical records to identify incident stroke events, continuing until the end of 2024 (Figure 1). The study was approved by the Ethics Committee of Shanghai East Hospital (Approval No: 2021YYS-203) in accordance with the Declaration of Helsinki, and informed consent was waived.

### 2.2 Data collection

All participants underwent standardized questionnaire assessments (including age, sex, smoking status, alcohol consumption, and TCM constitution identification) and anthropometric measurements (height, weight, systolic blood pressure, diastolic blood pressure), and body mass index (BMI) was calculated. Participants were required to fast for at least 8 h prior to venous blood sampling for laboratory tests, including complete blood count (red blood cells (RBC), white blood cells (WBC), lymphocytes, monocyte, neutrophils, platelets), hemoglobin, fasting plasma glucose (FPG), triglycerides (TG), total cholesterol (TC), HDL-C, low-density lipoprotein cholesterol (LDL-C), aspartate aminotransferase (AST), alanine aminotransferase (ALT), total bilirubin (TBIL), blood urea nitrogen (BUN), and serum creatinine (sCr). The estimated glomerular filtration rate (eGFR) was calculated using the CKD-EPI 2009 equation (34).

Furthermore, the major chronic conditions considered in this study included hypertension, type 2 diabetes mellitus (T2DM), dyslipidemia, CHD, stroke, heart failure (HF), arrhythmias (tachycardia/bradycardia, ventricular fibrillation, atrial fibrillation, etc.), chronic kidney disease (CKD), MAFLD, chronic obstructive pulmonary disease (COPD), and cancer. The presence of these chronic diseases was determined by medical staff based on electronic medical records.

### 2.3 TCM constitution assessment

The Constitution in Chinese Medicine Questionnaire (CCMQ), recommended by the China Association of Chinese Medicine, was used for standardized constitution identification (35). This assessment tool has been widely used in large-scale observational studies and community health interventions. Cross-culturally validated versions are available in Japanese, Korean, English, and Cantonese. A simplified version for older adults aged ≥65 years, the TCM Body constitution Questionnaire in elderly people (TCMECQ) has also been developed (36). Accordingly, uniformly trained healthcare professionals administered the TCMECQ for constitution identification in older adults. This study focused on the GTC and seven biased constitutions, including QDC, YaDC, YiDC, PDC, DHC, BSC, and QSC. Using the GTC as the reference, YaDC and DHC constitutions were associated with a higher risk of stroke and were therefore defined as “high-risk biased constitutions” for subsequent analysis (Supplementary Table S1).

### 2.4 Metaflammation indices

In this study, the LHR, MHR, NHR, and PHR were calculated using lymphocyte count (10^9^/L), monocyte count (10^9^/L), neutrophil count (10^9^/L), and platelet count (10^12^/L), respectively, divided by HDL-C (mmol/L).

### 2.5 Outcome

The primary outcome was the first occurrence of any stroke event (including ischemic and hemorrhagic stroke). Outcome events were identified through annual follow-up (via telephone or face-to-face interview) and cross-verified by linking to the institutional electronic medical database. Follow-up continued until December 31, 2024. All suspected events were confirmed by medical staffs based on ICD-10 diagnostic codes and detailed medical history.

### 2.6 Statistical analysis

The skewness and kurtosis test was used to assess the normality of continuous variables. Continuous variables were presented as mean ± standard deviation or median (interquartile range, IQR), and categorical variables as frequency (percentage). First, univariable Cox proportional hazards regression models were employed to preliminarily evaluate the associations between all baseline covariates (including demographic characteristics, clinical indicators, constitution types, and metaflammation indices) and stroke risk. This was used to screen covariates for inclusion in multivariable models (selecting those with *p* < 0.20 or of clear clinical importance). As detailed in Supplementary Table S1, the following variables were selected for adjustment after assessing multicollinearity: age, sex, CHD, arrhythmias, COPD, BMI, hemoglobin, and eGFR. Variables not meeting the pre-specified selection criterion (*p* < 0.20) and without clear clinical importance for stroke prediction in the elderly, such as LDL-C and hypertension, were not included in the final models to preserve statistical power and avoid overfitting given the limited number of outcome events (*n* = 36). Subsequently, multivariable Cox proportional hazards regression models were used to examine the total and independent associations of metaflammation indices (LHR, MHR, NHR, PHR) and constitution types with stroke risk. Furthermore, including all potential confounders in post-hoc exploratory models did not materially alter the effect estimates for LHR, PHR, or TCM constitutions (Supplementary Tables S5, S6). To examine the differential associations between metaflammation indices and stroke risk across different constitution types, stratified analyses were conducted within the Gentleness, low-risk biased constitution, and high-risk biased constitution subgroups. Multiplicative interaction between metaflammation indices and constitution type grouping was tested. Additionally, TCM constitutions were reclassified into a binary variable (GTC + low-risk biased constitutions vs. high-risk biased constitutions), and the aforementioned analyses were repeated. Three sensitivity analyses were conducted: (1) excluding participants with other baseline cardiovascular diseases (including coronary heart disease, heart failure, and arrhythmias); (2) excluding individuals who developed incident stroke in 2021; and (3) excluding both of the above categories of individuals. These steps were taken to examine the robustness of the associations between metaflammation indices and stroke risk, as well as the differences of these associations.

All analyses were performed using STATA 18.0 (StataCorp LLC, Texas, USA) and R 4.5.1 (R Foundation for Statistical Computing, Vienna, Austria). A two-sided *p*-value < 0.05 was considered statistically significant.

## 3 Results

### 3.1 Baseline characteristics of participants

This study ultimately included 2,999 community-dwelling older adults with a median age of 70 years (IQR: 68–74), and 41.61% were female. As detailed in Table 1, GTC was the most prevalent TCM constitution type (40.11%), followed by DHC (13.44%) and QDC (11.54%). Hypertension (65.56%), MAFLD (21.84%), CKD (18.01%), and T2DM (11.47%) were the most common comorbidities. The median values of the metaflammation indices, calculated from laboratory results, were as follows: LHR 1.45 (IQR: 1.10–1.89), MHR 0.23 (IQR: 0.17–0.31), NHR 2.29 (IQR: 1.72–3.05), and PHR 149.61 (IQR: 119.39–185.16). During the 4-year follow-up period, 36 incident stroke events occurred, yielding an incidence rate of 3.01 per 1,000 person-years.

**Table 1:** Baseline characteristics of participants (*N* = 2,999).

Variable	Total (*N* = 2,999)
Age (years), M (IQR)	70 (68–74)
Female, *n* (%)	1,248 (41.61)
Never smoked, *n* (%)	2,989 (99.67)
Never consumed alcohol, *n* (%)	2,985 (99.53)
TCM constitution, *n* (%)	
GTC	1,203 (40.11)
QDC	346 (11.54)
YaDC	172 (5.74)
YiDC	234 (7.80)
PDC	279 (9.30)
DHC	403 (13.44)
BSC	245 (8.17)
QSC	117 (3.90)
Comorbidity, *n* (%)	
Hypertension	1,966 (65.56)
T2DM	344 (11.47)
Dyslipidemia	119 (3.97)
CHD	111 (3.70)
Arrhythmias	5 (0.17)
HF	1 (0.03)
CKD	540 (18.01)
MAFLD	655 (21.84)
COPD	16 (0.53)
Cancer	0 (0.00)
Anthropometric measurements	
BMI (kg/m^2^), M (IQR)	22.60 (21.48–23.51)
SBP (mmHg), M (IQR)	124 (120–130)
DBP (mmHg), M (IQR)	75 (70–78)
Laboratory tests	
RBC (10^12/L), M (IQR)	4.7 (4.4–5.0)
Hemoglobin (g/L), M (IQR)	142 (133–151)
WBC (10^9/L), M (IQR)	5.9 (5.0–6.9)
Lymphocytes (10^9/L), M (IQR)	2.0 (1.7–2.5)
Monocytes (10^9/L), M (IQR)	0.3 (0.3–0.4)
Neutrophils (10^9/L), M (IQR)	3.2 (2.7–3.9)
Platelets (10^9/L), M (IQR)	213 (182–248)
FPG (mmol/L), M (IQR)	5.70 (5.20–6.60)
TG (mmol/L), M (IQR)	1.50 (1.13–2.00)
TC (mmol/L), M (IQR)	5.38 (4.64–6.18)
HDL-C (mmol/L), M (IQR)	1.41 (1.22–1.67)
LDL-C (mmol/L), M (IQR)	3.09 (2.48–3.68)
AST (U/L), M (IQR)	19 (14–26)
ALT (U/L), M (IQR)	19 (16–24)
TBIL (μmol/L), M (IQR)	11.9 (9.5–15.3)
BUN (mmol/L), M (IQR)	5.6 (4.8–6.6)
sCr (μmol/L), M (IQR)	70 (60–81)
eGFR (mL/min/1.73 m^2^), M (IQR)	86.8 (65.1–96.2)
Metaflammation indices	
LHR, M (IQR)	1.45 (1.10–1.89)
MHR, M (IQR)	0.23 (0.17–0.31)
NHR, M (IQR)	2.29 (1.72–3.05)
PHR, M (IQR)	149.61 (119.39–185.16)
Number of incident stroke, *n* (%)	36 (1.20)
Incidence rate (per 1,000 people-year)	3.01

TCM, Traditional Chinese Medicine; GTC, Gentleness constitution; QDC, Qi-deficiency constitution; YaDC, Yang-deficiency constitution; YiDC, Yin-deficiency constitution; PDC, Phlegm-dampness constitution; DHC, Damp-heat constitution; BSC, Blood-stasis constitution; QSC, Qi-stagnation constitution; T2DM, type 2 diabetes mellitus; CHD, coronary heart disease; HF, heart failure; CKD, chronic kidney disease; MAFLD, metabolic dysfunction-associated fatty liver disease; COPD, chronic obstructive pulmonary disease; BMI, body mass index; SBP, systolic blood pressure; DBP, diastolic blood pressure; RBC, red blood cell; WBC, white blood cell; FPG, fasting plasma glucose; TG, triglycerides; TC, total cholestero; HDL-C, high-density lipoprotein cholesterol; LDL-C, low-density lipoprotein cholesterol; AST, aspartate aminotransferase; ALT, alanine aminotransferase; TBIL, total bilirubin; BUN, blood urea nitrogen; sCr, serum creatinine; eGFR, estimated glomerular filtration rate; LHR, lymphocyte to high-density lipoprotein cholesterol ratio; MHR, monocyte to high-density lipoprotein cholesterol ratio; NHR, neutrophil to high-density lipoprotein cholesterol ratio; PHR, platelet to high-density lipoprotein cholesterol ratio; CI, confidence interval.

Notably, when comparing TCM constitutions (Supplementary Tables S2–S4), the high-risk biased constitution group (YaDC + DHC) exhibited a paradoxical pattern: despite having significantly higher prevalence of hypertension (70.43% vs. 60.85% in GTC, *p* < 0.001) and CHD (5.39% vs. 0.08% in GTC, *p* < 0.001), as well as higher BMI (22.86 vs. 22.31 kg/m^2^, *p* < 0.001), this group showed lower median levels of LHR (1.39 vs. 1.46, *p* = 0.036) and NHR (2.22 vs. 2.32, *p* = 0.039) compared to the GTC group. This inverse relationship between traditional cardiovascular risk burden and metaflammation indices in the high-risk constitution group suggests that the biological pathways driving stroke risk in these individuals may be distinct from those captured by LHR and PHR.

### 3.2 Total and independent effects of metaflammation indices and TCM constitution on incident stroke

Univariable Cox regression analysis (Supplementary Table S1) revealed that higher levels of LHR (HR = 1.76, 95% CI: 1.16–2.67, *p* = 0.007), NHR (HR = 1.30, 95% CI: 1.02–1.66, *p* = 0.036), and PHR (HR = 1.01, 95% CI: 1.00–1.01, *p* = 0.023) were significantly associated with an increased risk of stroke, while MHR showed no significant association (*p* = 0.822). Among the TCM constitution types, compared to the GTC, both YaDC (HR = 4.99, 95% CI: 1.58–15.73, *p* = 0.006) and DHC (HR = 3.00, 95% CI: 1.05–8.56, *p* = 0.040) were significantly associated with a higher stroke risk.

Multivariable Cox proportional hazards models (Table 2) showed that in the total effect models, LHR (HR = 1.68, 95% CI: 1.09–2.61, *p* = 0.020) and PHR (HR = 1.01, 95% CI: 1.00–1.01, *p* = 0.009) remained significantly and positively associated with incident stroke after adjustment for confounders. High-risk biased constitutions (including YaDC and DHC) were also significantly associated with elevated stroke risk compared to GTC (HR = 3.42, 95% CI: 1.32–8.80, *p* = 0.011). In the independent effect models, after mutual adjustment for metaflammation indices and constitution types, LHR, PHR, and high-risk biased constitutions (HR > 1, *p* < 0.05) all remained significantly associated with an increased risk of stroke, whereas MHR and NHR did not show statistical significance (*p* > 0.05).

**Table 2:** Total and independent effects of LHR, PHR, and TCM constitution on incident stroke using multivariable Cox proportional hazards models.

Variable	HR (95%CI)	*p*
Total effects^a^		
LHR	1.68 (1.09, 2.61)	0.020
MHR	1.06 (0.43, 2.60)	0.905
NHR	1.24 (0.94, 1.64)	0.123
PHR	1.01 (1.00, 1.01)	0.009
TCM constitution		
GTC (as reference)	/	/
Low-risk biased constitutions^c^	2.00 (0.81, 4.95)	0.135
High-risk biased constitutions^c^	3.42 (1.32, 8.80)	0.011
Independent effects^b^		
LHR	1.72 (1.11, 2.68)	0.016
TCM constitution		
GTC (as reference)	/	/
Low-risk biased constitutions^c^	1.91 (0.77, 4.73)	0.164
High-risk biased constitutions^c^	3.54 (1.37, 9.12)	0.009
MHR	1.07 (0.48, 2.41)	0.896
TCM constitution		
GTC (as reference)	/	/
Low-risk biased constitutions^c^	2.00 (0.81, 4.95)	0.135
High-risk biased constitutions^c^	3.42 (1.32, 8.81)	0.011
NHR	1.28 (0.96, 1.70)	0.088
TCM constitution		
GTC (as reference)	/	/
Low-risk biased constitutions^c^	1.99 (0.81, 4.92)	0.138
High-risk biased constitutions^c^	3.59 (1.39, 9.30)	0.008
PHR	1.01 (1.00, 1.01)	0.010
TCM constitution		
GTC (as reference)	/	/
Low-risk biased constitutions^c^	1.94 (0.78, 4.82)	0.154
High-risk biased constitutions^c^	3.48 (1.35, 8.98)	0.010

^a^The Cox proportional hazards models for estimating the total effects of LHR, MHR, NHR, PHR, and TCM constitution were adjusted for the following confounding variables: age, sex, CHD, arrhythmias, COPD, BMI, hemoglobin, and eGFR.

^b^The Cox proportional hazards models for estimating the independent effects of LHR, MHR, NHR, and PHR were adjusted for the following confounding variables: age, sex, CHD, arrhythmias, COPD, BMI, hemoglobin, eGFR, and TCM constitution.

^c^Low-risk biased constitutions included QDC, YiDC, PDC, BSC, and QSC; while high-risk biased constitutions included YaDC and DHC.

HR, hazard ratio; CI, confidence interval; LHR, lymphocyte to high-density lipoprotein cholesterol ratio; PHR, platelet to high-density lipoprotein cholesterol ratio; TCM, Traditional Chinese Medicine; GTC, Gentleness constitution; CHD, coronary heart disease; COPD, chronic obstructive pulmonary disease; BMI, body mass index; eGFR, estimated glomerular filtration rate; QDC, Qi-deficiency constitution; YiDC, Yin-deficiency constitution; PDC, Phlegm-dampness constitution; BSC, Blood-stasis constitution; QSC, Qi-stagnation constitution; YaDC, Yang-deficiency constitution; DHC, Damp-heat constitution.

### 3.3 Differential associations of metaflammation indices with incident stroke by TCM constitutions

Stratified analyses were conducted to examine the differential associations between metaflammation indices and stroke risk across different TCM constitution types (Table 3). The results indicated that the associations of LHR and PHR varied across constitution subgroups. Specifically, among individuals with the GTC, each unit increase in LHR was associated with a 137% increase in stroke risk (HR = 2.37, 95% CI: 1.03–5.48, *p* = 0.043). Among those with low-risk biased constitutions (including QDC, YiDC, PDC, BSC, and QSC), both LHR (HR = 1.99, 95% CI: 1.07–3.69, *p* = 0.029) and PHR (HR = 1.01, 95% CI: 1.00–1.01, *p* = 0.009) demonstrated a positive association with incident stroke. However, among individuals with high-risk biased constitutions (including YaDC and DHC), neither LHR (HR = 1.17, 95% CI: 0.42–3.22, *p* = 0.765) nor PHR (HR = 1.00, 95% CI: 0.99–1.02, *p* = 0.490) was significantly associated with stroke risk.

**Table 3:** Associations of LHR and PHR on incident stroke in different TCM constitutions using multivariable Cox proportional hazards models.

Subgroup	LHR^a^			PHR^a^		
	HR (95%CI)	*p*	*p* for interaction	HR (95%CI)	*p*	*p* for interaction
TCM constitution (three-category)			0.146			0.531
GTC	2.37 (1.03, 5.48)	0.043		1.01 (0.99, 1.02)	0.313	
Low-risk biased constitutions^b^	1.99 (1.07, 3.69)	0.029		1.01 (1.00, 1.01)	0.009	
High-risk biased constitutions^b^	1.17 (0.42, 3.22)	0.765		1.00 (0.99, 1.02)	0.490	
TCM constitution (binary)			0.184			0.229
GTC + low-risk biased constitutions^b^	1.99 (1.24, 3.20)	0.005		1.01 (1.00, 1.01)	0.004	
High-risk biased constitutions^b^	1.17 (0.42, 3.22)	0.765		1.00 (0.99, 1.02)	0.490	

^a^The Cox proportional hazards models for estimating the independent effects of LHR and PHR were adjusted for the following confounding variables: age, sex, CHD, arrhythmias, COPD, BMI, hemoglobin, eGFR, and TCM constitution.

^b^Low-risk biased constitutions included QDC, YiDC, PDC, BSC, and QSC; while high-risk biased constitutions included YaDC and DHC.

HR, hazard ratio; CI, confidence interval; LHR, lymphocyte to high-density lipoprotein cholesterol ratio; PHR, platelet to high-density lipoprotein cholesterol ratio; TCM, Traditional Chinese Medicine; GTC, Gentleness constitution; CHD, coronary heart disease; COPD, chronic obstructive pulmonary disease; BMI, body mass index; eGFR, estimated glomerular filtration rate; QDC, Qi-deficiency constitution; YiDC, Yin-deficiency constitution; PDC, Phlegm-dampness constitution; BSC, Blood-stasis constitution; QSC, Qi-stagnation constitution; YaDC, Yang-deficiency constitution; DHC, Damp-heat constitution.

This differential pattern persisted when constitution types were combined into a binary variable (GTC + low-risk biased constitutions vs. high-risk biased constitutions). The associations of LHR and PHR remained significant in the “GTC + low-risk biased constitutions” group (LHR: HR = 1.99, 95% CI: 1.24–3.20, *p* = 0.005; PHR: HR = 1.01, 95% CI: 1.00–1.01, *p* = 0.004) but were non-significant in the “high-risk biased constitutions” group (both *p* > 0.05). The *p*-values for interaction were 0.184 and 0.229 for LHR and PHR, respectively.

### 3.4 Sensitivity analyses

Three sensitivity analyses were performed to verify the robustness of the findings (Table 4).

**Table 4:** Associations of LHR and PHR on incident stroke in different TCM constitutions using multivariable Cox proportional hazards models (sensitivity analyses).

Total/subgroup	LHR^a^			PHR^a^		
	HR (95%CI)	*p*	*p* for interaction	HR (95%CI)	*p*	*p* for interaction
Sensitivity analysis 1						
TCM constitution (three-category)	1.93 (1.23, 3.03)	0.004	0.183	1.01 (1.00, 1.01)	0.035	0.650
GTC	2.37 (1.03, 5.48)	0.043		1.01 (0.99, 1.02)	0.313	
Low-risk biased constitutions^b^	2.37 (1.24, 4.54)	0.009		1.01 (1.00, 1.01)	0.077	
High-risk biased constitutions^b^	1.36 (0.49, 3.80)	0.552		1.01 (0.99, 1.02)	0.342	
TCM constitution (binary)	1.96 (1.25, 3.06)	0.003	0.147	1.01 (1.00, 1.01)	0.033	0.353
GTC + low-risk biased constitutions^b^	2.18 (1.35, 3.52)	0.001		1.01 (1.00, 1.01)	0.029	
High-risk biased constitutions^b^	1.36 (0.49, 3.80)	0.552		1.01 (0.99, 1.02)	0.342	
Sensitivity analysis 2						
TCM constitution (three-category)	1.63 (0.90, 2.95)	0.105	0.155	1.00 (0.99, 1.01)	0.638	0.385
GTC	1.53 (0.52, 4.57)	0.447		1.00 (0.99, 1.02)	0.565	
Low-risk biased constitutions^b^	3.22 (1.24, 8.31)	0.016		1.00 (0.99, 1.01)	0.649	
High-risk biased constitutions^b^	1.01 (0.30, 3.40)	0.983		1.00 (0.99, 1.02)	0.954	
TCM constitution (binary)	1.63 (0.90, 2.95)	0.105	0.122	1.00 (0.99, 1.01)	0.638	0.323
GTC + low-risk biased constitutions^b^	2.05 (1.08, 3.88)	0.027		1.00 (0.99, 1.01)	0.387	
High-risk biased constitutions^b^	1.01 (0.30, 3.40)	0.983		1.00 (0.99, 1.02)	0.954	
Sensitivity analysis 3						
TCM constitution (three-category)	1.81 (1.00, 3.25)	0.048	0.246	1.00 (0.99, 1.01)	0.500	0.532
GTC	1.53 (0.51, 4.57)	0.447		1.00 (0.99, 1.02)	0.565	
Low-risk biased constitutions^b^	3.25 (1.28, 8.27)	0.013		1.00 (0.99, 1.01)	0.680	
High-risk biased constitutions^b^	1.26 (0.37, 4.34)	0.713		1.00 (0.99, 1.02)	0.710	
TCM constitution (binary)	1.81 (1.01, 3.25)	0.048	0.146	1.00 (0.99, 1.01)	0.500	0.417
GTC + low-risk biased constitutions^b^	2.08 (1.11, 3.92)	0.023		1.00 (1.00, 1.01)	0.368	
High-risk biased constitutions^b^	1.26 (0.37, 4.34)	0.713		1.00 (0.99, 1.02)	0.710	

^a^The Cox proportional hazards models for estimating the independent effects of LHR and PHR were adjusted for the following confounding variables: age, sex, CHD, arrhythmias, COPD, BMI, hemoglobin, eGFR, and TCM constitution.

^b^Low-risk biased constitutions included QDC, YiDC, PDC, BSC, and QSC; while high-risk biased constitutions included YaDC and DHC.

HR, hazard ratio; CI, confidence interval; LHR, lymphocyte to high-density lipoprotein cholesterol ratio; PHR, platelet to high-density lipoprotein cholesterol ratio; TCM, Traditional Chinese Medicine; GTC, Gentleness constitution; CHD, coronary heart disease; COPD, chronic obstructive pulmonary disease; BMI, body mass index; eGFR, estimated glomerular filtration rate; QDC, Qi-deficiency constitution; YiDC, Yin-deficiency constitution; PDC, Phlegm-dampness constitution; BSC, Blood-stasis constitution; QSC, Qi-stagnation constitution; YaDC, Yang-deficiency constitution; DHC, Damp-heat constitution.

After excluding participants with baseline other cardiovascular diseases (CHD, HF, arrhythmias), the associations of LHR with stroke risk remained significant in the overall population (HR = 1.93, 95% CI: 1.23–3.03, *p* = 0.004), the GTC subgroup (HR = 2.37, 95% CI: 1.03–5.48, *p* = 0.043), and the low-risk biased constitutions subgroup (HR = 2.37, 95% CI: 1.24–4.54, *p* = 0.009). Similar results were observed when grouping by the binary constitution variable. No significant association was observed for LHR in the high-risk biased constitutions subgroup (*p* > 0.05). The associations for PHR across subgroups showed similar patterns, although no significant interaction between constitution type and LHR or PHR was detected in this analysis.

After excluding incident stroke cases occurring in 2021 to minimize reverse causality, a significant association between LHR and increased stroke risk was observed only in the “low-risk biased constitutions” subgroup (HR = 3.22, 95% CI: 1.24–8.31, *p* = 0.016) and the “GTC + low-risk biased constitutions” subgroup (HR = 2.05, 95% CI: 1.08–3.88, *p* = 0.027), but not in the high-risk biased constitutions subgroup.

After simultaneously excluding baseline CVD and 2021 incident stroke cases, LHR remained associated with increased stroke risk in the overall population, the “low-risk biased constitutions” subgroup, and the “GTC + low-risk biased constitutions” subgroup.

Furthermore, as shown in Supplementary Table S7, high-risk biased constitutions were consistently and significantly associated with an elevated risk of stroke (HR > 1, *p* < 0.05) across sensitivity analyses.

## 4 Discussion

This study is the first to examine the associations of new inflammation indices with incident stroke in a community-based cohort and analyze their differences across TCM constitution types. The main findings: LHR and PHR can predict stroke in older adults; TCM constitution types, particularly YaDC and DHC, are important stroke risk factors; and the strength of the associations between inflammation indices and stroke varies by TCM type. Significant associations were observed in individuals with GTC and low-risk constitutions, but not in those with YaDC or DHC.

LHR integrates information from lymphocytes (a marker of immunoregulation and potential inflammaging) (9, 37) and HDL-C (which has anti-inflammatory, antioxidant, and reverse cholesterol transport functions) (20, 38). PHR reflects the balance between platelets (involved in atherosclerotic thrombosis and inflammation) (39, 40) and HDL-C function. The lack of a significant association for MHR, despite the well-recognized role of monocytes in metaflammation (41, 42), may be explained by the lower dynamic range or signal-to-noise ratio of monocyte counts in this relatively healthy, community-dwelling elderly cohort with a low event rate. In contrast, lymphocytes and platelets exhibit greater sensitivity to systemic inflammatory and stress responses—processes that may precede a cerebrovascular event. Specifically, LHR captures the balance between adaptive immune cells (primarily T- and B-lymphocytes), which are key drivers of “inflammaging” and chronic inflammation, and HDL-C’s anti-inflammatory and antioxidant functions. Growing evidence suggests that HDL-C can functionally modulate immune cell activation; dysfunctional HDL may lose its anti-inflammatory capacity and even acquire pro-inflammatory properties (15, 20, 43, 44). This “HDL quality” rather than ‘HDL quantity’ perspective underscores the functional significance of HDL-C in the context of metaflammation. Consequently, the LHR- and PHR-integrated balance may be more relevant than monocyte-driven pathways in reflecting low-grade, sustained metaflammation that contributes to stroke risk in older adults (45).

The results reinforce the importance of TCM constitution types as endogenous factors for stroke risk. We found that individuals with YaDC and DHC had a significantly higher risk of stroke compared to those with GTC—a finding broadly consistent with prior reports of elevated stroke risk in these constitutions (46, 47), though the specific high-risk phenotypes identified vary across studies owing to regional and sociocultural differences. This association likely reflects the accumulation of cardiometabolic risk factors (26–31). Intriguingly, as shown in Supplementary Tables S2–S4, despite having a higher burden of hypertension, CHD, and BMI, individuals with high-risk biased constitutions paradoxically exhibited lower median levels of LHR, NHR, and PHR compared to those with GTC. This “inflammatory paradox” suggests that the pathophysiological pathways driving stroke risk in high-risk constitutions may be distinct from the metaflammation axis reflected by these composite indices.

To substantiate this hypothesis with concrete signaling frameworks, we propose a unified model in which constitution-dependent predictive divergence arises from differential immune-metabolic remodeling at three interlocked levels. First, at the tissue macrophage level, Galectin-9 has been shown in the tumor microenvironment to bind Tim-3 to recruit PI3K-p85 and activate the PI3K/Akt pathway, driving macrophages toward an M2-like phenotype (characterized by upregulated arginase-1, CD163, and IL-10, and downregulated iNOS) (48). Extending this mechanism to the vascular context, we hypothesize that high-risk biased constitutions (YaDC/DHC) may already reside in a state of sustained, Galectin-9–mediated M2 immune tolerance in the vascular wall; this local remodeling would drive stroke risk through tissue-predominant mechanisms that are uncoupled from circulating lymphocyte and platelet counts, directly accounting for the observed “inflammatory paradox” wherein LHR and PHR are paradoxically lower and non-predictive despite higher clinical risk burden. In contrast, balanced constitutions lack this fixed polarized state, allowing circulating LHR and PHR to sensitively reflect early systemic metaflammation. Second, at the intracellular redox level, oxidative stress reflects a disturbance in cellular redox balance where ROS and reactive nitrogen species function as signaling mediators within the multilayered antioxidant defense network (SOD, CAT, GPX, GSH) (49). Constitution-specific redox set-points—particularly differential baseline NRF2/KEAP1 activity—may create divergent inflammatory thresholds: in balanced constitutions, an elevated LHR may signal an early deviation from redox homeostasis before structural damage ensues, whereas in high-risk constitutions, chronic pathway activation may exhaust adaptive antioxidant capacity, shifting immune cells toward a tolerogenic, pro-remodeling phenotype that renders circulating metaflammation indices uninformative. Third, at the systemic immune level, machine-learning analyses across cancers, metabolic, respiratory, and digestive diseases reveal that CD4+ T cells operate as context-dependent immune-metabolic sensors via distinct transcriptional programs (AK4, CALU, LINC01271 signatures) (50). Extending this concept, we posit that TCM constitutions represent stable transcriptional endotypes with distinct redox-sensitive signaling thresholds. Under this model, LHR and PHR would be expected to reflect early, dynamic T-cell–mediated systemic shifts in low-risk constitutions, whereas in high-risk constitutions, fixed tissue-level remodeling—driven by polarized macrophage phenotypes and exhausted redox adaptation—may dominate stroke risk without elevating circulating metaflammation indices.

A core observation of this study is the presence of constitution-dependent variations in the associations between metaflammation indices and stroke risk, although formal interaction tests were not statistically significant (*p* for interaction > 0.05). These variations align with the hypothesis that distinct TCM constitutions harbor different intrinsic pathophysiological profiles (24, 25), gene expression backgrounds (51), and the immune-metabolic remodeling mechanisms proposed above. However, because the interaction tests were non-significant, this integrated mechanistic framework remains hypothesis-generating. Validation through longitudinal biomarker trajectories and constitution-stratified experimental models (e.g., monocyte/macrophage polarization assays, single-cell transcriptomics, or redox profiling) is therefore warranted before clinical translation. Nevertheless, the present findings offer immediate implications for personalized prevention: individuals with high-risk constitutions (e.g., YaDC, DHC) should be regarded as a high-risk group warranting active primary prevention regardless of their current metaflammation index levels, as their stroke risk appears driven by tissue-level remodeling that circulating indices may not capture. For individuals with GTC or low-risk constitutions, indicators like LHR and PHR could serve as dynamic monitoring tools to identify those transitioning from a stable to an inflammatory-metabolically disturbed state, allowing for timely intervention. Future stroke risk prediction models might consider integrating TCM constitution typing with traditional risk factors and novel biomarkers to build a more refined risk stratification system.

This study has several important limitations that should be considered. First and foremost, the low number of incident stroke events (*n* = 36) raises concerns about statistical power and model overfitting, making our estimates, particularly in subgroup analyses, potentially unstable. Consequently, our findings are exploratory and require replication in larger cohorts. Second, although a cohort design was used, both the metaflammation indices and TCM constitution assessments were conducted only at baseline, preventing the observation of their dynamic changes over time and limiting causal inference. Third, the observed stroke incidence rate in our cohort (3.01 per 1,000 person-years) is comparable to the national age-standardized incidence of approximately 205 per 100,000 person-years for adults in China (55). Considering that our cohort exclusively comprises individuals aged ≥65 years, the observed rate is within an expected range. However, modest differences may be attributable to healthy survivor bias, as participants were volunteers in an annual health check-up program and tend to be healthier than the general elderly population, as well as potential under-ascertainment of mild, non-hospitalized strokes from electronic medical records. Fourth, despite adjusting for multiple important confounders, the possibility of residual confounding remains—particularly from unmeasured lifestyle factors such as detailed dietary intake, physical activity, and sleep quality, as well as from medication use (e.g., statins, antihypertensive drugs, antiplatelet agents), for which reliable data were not available in this retrospective database. Fifth, data on coagulation markers (e.g., fibrinogen, D-dimer, INR, aPTT) were not collected, as they are not part of routine community-based annual health check-ups for asymptomatic older adults. The absence of these well-established stroke risk factors precludes adjustment for their potential confounding effects or exploration of interactions with platelet-related indices. Furthermore, the lack of data on neurological function, the microbiome-gut-brain axis, or related biomarkers (e.g., short-chain fatty acids) prevents us from exploring the mechanistic pathways linking hypertension, metaflammation, and stroke—an important avenue for future research. Finally, the generalizability of our findings to other regions, ethnicities, or younger populations requires further validation.

## 5 Conclusion

In summary, this community-based cohort study demonstrates that elevated LHR and PHR are independent risk factors for incident stroke in the elderly. Furthermore, TCM constitution types, specifically Yang-deficiency and Damp-heat constitutions, significantly modify the associations between these metaflammation indices and stroke risk. The predictive utility of LHR and PHR was significant only in individuals with Gentleness or low-risk biased constitutions, but not in those with high-risk biased constitutions. These findings highlight the value of integrating TCM constitution theory with modern inflammatory-metabolic biomarkers for achieving a more personalized and precise risk stratification in stroke prevention. Future research should validate these findings in larger, more diverse populations and explore the underlying mechanisms linking specific constitutions to metaflammation and stroke vulnerability.

## Data Availability

The raw data supporting the conclusions of this article will be made available by the authors, without undue reservation.
