# Supplementary material for: Differential associations of novel metaflammation indices with incident stroke across traditional Chinese medicine constitutions: a retrospective community-based cohort study of older adults
Source: Front Neurol. 2026 Jun 3;17:1750848. doi: 10.3389/fneur.2026.1750848 (PMC13272024; doi:10.3389/fneur.2026.1750848)
Supplement: Supplementary file 1 [file Table_1.DOCX]

**Table S1 Covariate selection using univariable Cox proportional hazards model**

|  | HR(95%CI) | *P* |
| --- | --- | --- |
| Age | 1.06(1.01,1.11) | 0.021 |
| Female (vs. male) | 1.57(0.82,3.02) | 0.176 |
| Ever smoked (vs. never) | / | / |
| Ever consumed alcohol (vs. never) | / | / |
| TCM constitution |  |  |
| GTC (as reference) | 1 | / |
| QDC | 2.49(0.79,7.85) | 0.119 |
| YaDC | 4.99(1.58,15.73) | 0.006 |
| YiDC | 2.95(0.86,10.08) | 0.084 |
| PDC | 2.47(0.72,8.45) | 0.149 |
| DHC | 3.00(1.05,8.56) | 0.040 |
| BSC | 1.41(0.29,6.78) | 0.669 |
| QSC | 2.97(062,14.28) | 0.175 |
| Hypertension (vs. No) | 1.37(0.66,2.84) | 0.398 |
| T2DM (vs. No) | 0.45(0.11,1.87) | 0.277 |
| Dyslipidemia (vs. No) | 0.69(0.09,5.06) | 0.717 |
| CHD (vs. No) | 3.31(1.17,9.37) | 0.024 |
| Arrhythmias (vs. No) | 17.05(2.34,124.46) | 0.005 |
| HF (vs. No) | / | / |
| CKD (vs. No) | 1.10(0.48,2.51) | 0.820 |
| MAFLD (vs. No) | 0.58(0.22,1.48) | 0.253 |
| COPD (vs. No) | 5.49(0.75,40.08) | 0.093 |
| BMI | 1.10(1.00,1.21) | 0.063 |
| SBP | 1.01(0.97,1.05) | 0.660 |
| DBP | 1.03(0.97,1.10) | 0.272 |
| RBC | 1.60(0.81,3.18) | 0.175 |
| Hemoglobin | 1.02(1.00,1.04) | 0.107 |
| WBC | 1.18(1.05,1.34) | 0.007 |
| Lymphocytes | 1.66(1.15,2.41) | 0.007 |
| Monocytes | 1.03(0.57,1.84) | 0.929 |
| Neutrophils | 1.27(0.97,1.65) | 0.079 |
| Platelets | 1.00(1.00,1.01) | 0.095 |
| FPG | 1.07(0.91,1.26) | 0.439 |
| TG | 0.97(0.68,1.37) | 0.846 |
| TC | 0.83(0.62,1.12) | 0.220 |
| HDL-C | 0.38(0.13,1.14) | 0.085 |
| LDL-C | 0.87(0.60,1.27) | 0.482 |
| AST | 1.00(0.99,1.01) | 0.959 |
| ALT | 0.99(0.95,1.03) | 0.564 |
| TBIL | 1.02(0.96,1.08) | 0.483 |
| BUN | 1.07(0.89,1.28) | 0.483 |
| sCr | 1.00(1.00,1.01) | 0.463 |
| eGFR | 0.99(0.97,1.00) | 0.092 |
| LHR | 1.76(1.16,2.67) | 0.007 |
| MHR | 1.06(0.63,1.79) | 0.822 |
| NHR | 1.30(1.02,1.66) | 0.036 |
| PHR | 1.10(1.00,1.01) | 0.023 |

Abbreviation: HR, hazard ratio;CI, confidence interval; TCM, Traditional Chinese Medicine; GTC, Gentleness constitution; QDC, Qi-deficiency constitution; YaDC, Yang-deficiency constitution; YiDC, Yin-deficiency constitution; PDC, Phlegm-dampness constitution; DHC, Damp-heat constitution; BSC, Blood-stasis constitution; QSC, Qi-stagnation constitution; T2DM, type 2 diabetes mellitus; CHD, coronary heart disease; HF, heart failure; CKD, chronic kidney disease; MAFLD, metabolic dysfunction-associated fatty liver disease; COPD, chronic obstructive pulmonary disease; BMI, body mass index; SBP, systolic blood pressure; DBP, diastolic blood pressure; RBC, red blood cell; WBC, white blood cell; FPG, fasting plasma glucose;TG, triglycerides; TC, total cholestero; HDL-C, high-density lipoprotein cholesterol; LDL-C, low-density lipoprotein cholesterol;AST, aspartate aminotransferase; ALT, alanine aminotransferase; TBIL, total bilirubin; BUN, blood urea nitrogen; sCr, serum creatinine; eGFR, estimated glomerular filtration rate; LHR, lymphocyte to high-density lipoprotein cholesterol ratio; MHR, monocyte to high-density lipoprotein cholesterol ratio; NHR, neutrophil to high-density lipoprotein cholesterol ratio; PHR, platelet to high-density lipoprotein cholesterol ratio.

**Table S2 Baseline characteristics of participants across TCM Constitutions (N=2999)**

|  | GTC  (N=1203) | QDC  (N=346) | YaDC  (N=172) | YiDC  (N=234) | PDC  (N=279) | DHC  (N=403) | BSC  (N=245) | QSC  (N=117) |
| --- | --- | --- | --- | --- | --- | --- | --- | --- |
| Number of incident stroke, n(%) | 7(0.58) | 5(1.45) | 5(2.91) | 4(1.71) | 4(1.43) | 7(1.74) | 2(0.82) | 2(1.71) |
| Age (years), M(IQR) | 70.00(67.00-74.00) | 71.00(68.00-75.00) | 70.00(67.00-74.00) | 71.00(69.00-74.00) | 70.00(67.00-74.00) | 70.00(67.00-74.00) | 71.00(68.00-75.00) | 70.00(67.00-73.00) |
| Female, n (%) | 515(42.81) | 113(32.66) | 63(36.63) | 85(36.32) | 140(50.18) | 177(43.92) | 116(47.35) | 39(33.33) |
| BMI (kg/m^2^), M(IQR) | 22.31(21.26-23.03) | 22.77(21.48-23.83) | 22.86(21.81-23.44) | 22.60(21.48-23.88) | 23.23(22.49-24.22) | 22.86(21.48-24.00) | 23.03(22.09-24.03) | 22.49(21.48-23.66) |
| Hypertension, n (%) | 732(60.85) | 253(73.12) | 104(60.47) | 144(61.54) | 177(63.44) | 301(74.69) | 170(69.39) | 85(72.65) |
| T2DM, n (%) | 120(9.98) | 42(12.14) | 19(11.05) | 45(19.23) | 17(6.09) | 52(12.90) | 32(13.06) | 17(14.53) |
| CHD, n (%) | 1(0.08) | 20(5.78) | 3(1.74) | 12(5.13) | 5(1.79) | 28(6.95) | 38(15.51) | 4(3.42) |
| LHR, M(IQR) | 1.46(1.10-1.87) | 1.43(1.07-1.87) | 1.38(1.12-1.91) | 1.46(1.12-1.91) | 1.48(1.18-1.96) | 1.39(1.02-1.84) | 1.47(1.11-1.96) | 1.46(1.16-1.85) |
| MHR, M(IQR) | 0.23(0.17-0.31) | 0.23(0.17-0.32) | 0.24(0.17-0.32) | 0.23(0.17-0.32) | 0.25(0.19-0.33) | 0.22(0.16-0.30) | 0.24(0.18-0.33) | 0.23(0.18-0.30) |
| NHR, M(IQR) | 2.32(1.74-2.99) | 2.26(1.69-3.13) | 2.27(1.70-3.02) | 2.25(1.71-3.13) | 2.32(1.75-3.26) | 2.19(1.64-3.01) | 2.36(1.83-3.14) | 2.27(1.82-3.02) |
| PHR, M(IQR) | 148.46(120.81-185.16) | 151.37(115.24-189.42) | 152.42(114.49-189.10) | 151.21(119.79-182.50) | 152.38(118.45-182.65) | 145.45(115.15-181.46) | 152.66(121.43-185.37) | 158.26(135.20-192.92) |

Abbreviation: TCM, Traditional Chinese Medicine; GTC, Gentleness constitution; QDC, Qi-deficiency constitution; YaDC, Yang-deficiency constitution; YiDC, Yin-deficiency constitution; PDC, Phlegm-dampness constitution; DHC, Damp-heat constitution; BSC, Blood-stasis constitution; QSC, Qi-stagnation constitution; BMI, body mass index; T2DM, type 2 diabetes mellitus; CHD, coronary heart disease; LHR, lymphocyte to high-density lipoprotein cholesterol ratio; MHR, monocyte to high-density lipoprotein cholesterol ratio; NHR, neutrophil to high-density lipoprotein cholesterol ratio; PHR, platelet to high-density lipoprotein cholesterol ratio.

**Table S3 Baseline characteristics of participants across TCM Constitution types (N=2999)**

|  | GTC  (N=1203) | Low-risk biased constitutions  (N=1221) | High-risk biased constitutions  (N=575) | *P* |
| --- | --- | --- | --- | --- |
| Number of incident stroke, n(%) | 7(0.58) | 17(1.39) | 12(2.09) | 0.018 |
| Age (years), M(IQR) | 70.00(67.00-74.00) | 71.00(68.00-74.00) | 70.00(67.00-74.00) | 0.036 |
| Female, n (%) | 515(42.81) | 493(40.38) | 240(41.74) | 0.477 |
| BMI (kg/m^2^), M(IQR) | 22.31(21.26-23.03) | 22.86(21.83-24.03) | 22.86(21.48-23.66) | <0.001 |
| Hypertension, n (%) | 732(60.85) | 829(67.90) | 405(70.43) | <0.001 |
| T2DM, n (%) | 120(9.98) | 153(12.53) | 71(12.35) | 0.109 |
| CHD, n (%) | 1(0.08) | 79(6.47) | 31(5.39) | <0.001 |
| LHR, M(IQR) | 1.46(1.10-1.87) | 1.46(1.12-1.92) | 1.39(1.06-1.86) | 0.036 |
| MHR, M(IQR) | 0.23(0.17-0.31) | 0.23(0.18-0.32) | 0.23(0.17-0.30) | 0.064 |
| NHR, M(IQR) | 2.32(1.74-2.99) | 2.28(1.75-3.14) | 2.22(1.65-3.01) | 0.039 |
| PHR, M(IQR) | 148.46(120.81-185.16) | 152.63(120.00-185.71) | 147.37(114.89-184.16) | 0.209 |

Abbreviation: TCM, Traditional Chinese Medicine; BMI, body mass index; T2DM, type 2 diabetes mellitus; CHD, coronary heart disease; LHR, lymphocyte to high-density lipoprotein cholesterol ratio; MHR, monocyte to high-density lipoprotein cholesterol ratio; NHR, neutrophil to high-density lipoprotein cholesterol ratio; PHR, platelet to high-density lipoprotein cholesterol ratio.

**Table S4 Baseline characteristics of participants across TCM Constitution type (N=2999)**

|  | GTC+ Low-risk biased constitutions  (N=2424) | High-risk biased constitutions  (N=575) | *P* |
| --- | --- | --- | --- |
| Number of incident stroke, n(%) | 24(0.99) | 12(2.09) | 0.030 |
| Age (years), M(IQR) | 70.00(68.00-74.00) | 70.00(67.00-74.00) | 0.507 |
| Female, n (%) | 1008(41.58) | 240(41.74) | 0.946 |
| BMI (kg/m^2^), M(IQR) | 22.56(21.48-23.51) | 22.86(21.48-23.66) | 0.025 |
| Hypertension, n (%) | 1561(64.40) | 405(70.43) | 0.006 |
| T2DM, n (%) | 273(11.26) | 71(12.35) | 0.463 |
| CHD, n (%) | 80(3.30) | 31(5.39) | 0.017 |
| LHR, M(IQR) | 1.46(1.11-1.89) | 1.39(1.06-1.86) | 0.016 |
| MHR, M(IQR) | 0.23(0.18-0.32) | 0.23(0.17-0.30) | 0.025 |
| NHR, M(IQR) | 2.30(1.74-3.07) | 2.22(1.65-3.01) | 0.019 |
| PHR, M(IQR) | 150.85(120.57-185.38) | 147.37(114.89-184.16) | 0.086 |

Abbreviation: TCM, Traditional Chinese Medicine; BMI, body mass index; T2DM, type 2 diabetes mellitus; CHD, coronary heart disease; LHR, lymphocyte to high-density lipoprotein cholesterol ratio; MHR, monocyte to high-density lipoprotein cholesterol ratio; NHR, neutrophil to high-density lipoprotein cholesterol ratio; PHR, platelet to high-density lipoprotein cholesterol ratio.

**Table S5 Total and independent effects of LHR, PHR, and TCM constitution on incident stroke using multivariable Cox proportional hazards models adjusted for all confounders**

|  | HR(95%CI) | *P* |
| --- | --- | --- |
| **Total effects ^a^** |  |  |
| LHR | 1.71(1.09,2.67) | 0.019 |
| MHR | 1.05(0.39,2.80) | 0.921 |
| NHR | 1.26(0.94,1.68) | 0.122 |
| PHR | 1.01(1.00,1.01) | 0.005 |
| TCM constitution |  |  |
| GTC (as reference) | / | / |
| Low-risk biased constitutions ^c^ | 2.25(0.91,5.56) | 0.077 |
| High-risk biased constitutions ^c^ | 3.86(1.49,9.99) | 0.005 |
| **Independent effects ^b^** |  |  |
| LHR | 1.76(1.12,2.76) | 0.014 |
| TCM constitution |  |  |
| GTC (as reference) | / | / |
| Low-risk biased constitutions ^c^ | 2.19(0.89,5.41) | 0.089 |
| High-risk biased constitutions ^c^ | 4.08(1.57,10.59) | 0.004 |
| MHR | 1.06(0.46,2.47) | 0.885 |
| TCM constitution |  |  |
| GTC (as reference) | / | / |
| Low-risk biased constitutions ^c^ | 2.25(0.91,5.55) | 0.078 |
| High-risk biased constitutions ^c^ | 3.86(1.49,10.00) | 0.005 |
| NHR | 1.30(0.97,1.75) | 0.081 |
| TCM constitution |  |  |
| GTC (as reference) | / | / |
| Low-risk biased constitutions ^c^ | 2.26(0.91,5.57) | 0.077 |
| High-risk biased constitutions ^c^ | 4.09(1.57,10.64) | 0.004 |
| PHR | 1.01(1.00,1.01) | 0.004 |
| TCM constitution |  |  |
| GTC (as reference) | / | / |
| Low-risk biased constitutions ^c^ | 2.20(0.89,5.44) | 0.087 |
| High-risk biased constitutions ^c^ | 4.00(1.54,10.37) | 0.004 |

1. The Cox proportional hazards models for estimating the total effects of LHR, PHR, and TCM constitution were adjusted for the following confounding variables: age, sex, Hypertension, T2DM, Dyslipidemia, CHD, Arrhythmias, HF, CKD, MAFLD, COPD, Cancer, BMI, hemoglobin, FPG, LDL-C and eGFR.
2. The Cox proportional hazards models for estimating the independent effects of LHR and PHR were adjusted for the following confounding variables: age, sex, Hypertension, T2DM, Dyslipidemia, CHD, Arrhythmias, HF, CKD, MAFLD, COPD, Cancer, BMI, hemoglobin, FPG, LDL-C, eGFR, and TCM constitution.
3. Low-risk biased constitutions included QDC, YiDC, PDC, BSC, and QSC; while high-risk biased constitutions included YaDC and DHC.

**Table S6 Associations of LHR and PHR on incident stroke in different TCM constitutions using multivariable Cox proportional hazards models**

|  | LHR ^a^ | | | PHR ^a^ | | |
| --- | --- | --- | --- | --- | --- | --- |
|  | HR(95%CI) | *P* | *P* for interaction | HR(95%CI) | *P* | *P* for interaction |
| TCM constitution (three-category) |  |  | 0.176 |  |  | 0.570 |
| GTC | 2.82(1.25,6.37) | 0.012 |  | 1.01(0.99,1.02) | 0.311 |  |
| Low-risk biased constitutions ^b^ | 2.17(1.17,4.03) | 0.014 |  | 1.01(1.00,1.01) | 0.007 |  |
| High-risk biased constitutions ^b^ | 1.00(0.35,2.85) | 0.995 |  | 1.00(0.99,1.01) | 0.694 |  |
| TCM constitution (binary) |  |  | 0.205 |  |  | 0.275 |
| GTC + low-risk biased constitutions ^b^ | 2.08(1.29,3.36) | 0.005 |  | 1.01(1.00,1.01) | 0.002 |  |
| High-risk biased constitutions ^b^ | 1.00(0.35,2.85) | 0.995 |  | 1.00(0.99,1.01) | 0.694 |  |

1. The Cox proportional hazards models for estimating the independent effects of LHR and PHR were adjusted for the following confounding variables: age, sex, Hypertension, T2DM, Dyslipidemia, CHD, Arrhythmias, HF, CKD, MAFLD, COPD, Cancer, BMI, hemoglobin, FPG, LDL-C, eGFR, and TCM constitution.
2. Low-risk biased constitutions included QDC, YiDC, PDC, BSC, and QSC; while high-risk biased constitutions included YaDC and DHC.

**Table S7 Associations of TCM constitutions with incident stroke using multivariable Cox proportional hazards models (sensitivity analyses)**

|  | LHR ^a^ | | PHR ^b^ | |
| --- | --- | --- | --- | --- |
|  | HR(95%CI) | *P* | HR(95%CI) | *P* |
| Sensitivity analysis 1 |  |  |  |  |
| TCM constitution |  |  |  |  |
| GTC (as reference) | / | / | / | / |
| Low-risk biased constitutions ^c^ | 1.84(0.73,4.65) | 0.200 | 1.91(0.75,4.84) | 0.172 |
| High-risk biased constitutions ^c^ | 3.78(1.45,9.84) | 0.006 | 3.66(1.41,9.54) | 0.008 |
| TCM constitution (binary) |  |  |  |  |
| GTC + low-risk biased constitutions ^c^ | / | / | / | / |
| High-risk biased constitutions ^c^ | 2.66(1.26,5.61) | 0.010 | 2.52(1.20,5.30) | 0.014 |
| Sensitivity analysis 2 |  |  |  |  |
| TCM constitution |  |  |  |  |
| GTC (as reference) | / | / | / | / |
| Low-risk biased constitutions ^c^ | 0.95(0.30,3.01) | 0.936 | 0.99(0.31,3.12) | 0.986 |
| High-risk biased constitutions ^c^ | 3.57(1.24,10.25) | 0.018 | 3.48(1.21,9.99) | 0.021 |
| TCM constitution (binary) |  |  |  |  |
| GTC + low-risk biased constitutions ^c^ | / | / | / | / |
| High-risk biased constitutions ^c^ | 3.65(1.51,8.84) | 0.004 | 3.50(1.45,8.44) | 0.005 |
| Sensitivity analysis 3 |  |  |  |  |
| TCM constitution |  |  |  |  |
| GTC (as reference) | / | / | / | / |
| Low-risk biased constitutions ^c^ | 1.05(0.33,3.30) | 0.933 | 1.10(0.35,3.45) | 0.873 |
| High-risk biased constitutions ^c^ | 3.43(1.17,10.07) | 0.025 | 3.32(1.13,9.74) | 0.029 |
| TCM constitution (binary) |  |  |  |  |
| GTC + low-risk biased constitutions ^c^ | / | / | / | / |
| High-risk biased constitutions ^c^ | 3.35(1.34,8.35) | 0.010 | 3.17(1.28,7.89) | 0.013 |

1. The Cox proportional hazards models for estimating the independent effects of TCM constitutions were adjusted for the following confounding variables: age, sex, CHD, arrhythmias, COPD, BMI, hemoglobin, eGFR, and LHR.
2. The Cox proportional hazards models for estimating the independent effects of TCM constitutions were adjusted for the following confounding variables: age, sex, CHD, arrhythmias, COPD, BMI, hemoglobin, eGFR, and PHR.
3. Low-risk biased constitutions included QDC, YiDC, PDC, BSC, and QSC; while high-risk biased constitutions included YaDC and DHC.

Abbreviation: HR, hazard ratio;CI, confidence interval; LHR, lymphocyte to high-density lipoprotein cholesterol ratio; PHR, platelet to high-density lipoprotein cholesterol ratio; TCM, Traditional Chinese Medicine; GTC, Gentleness constitution; CHD, coronary heart disease; COPD, chronic obstructive pulmonary disease; BMI, body mass index; eGFR, estimated glomerular filtration rate; QDC, Qi-deficiency constitution; YiDC, Yin-deficiency constitution; PDC, Phlegm-dampness constitution; BSC, Blood-stasis constitution; QSC, Qi-stagnation constitution; YaDC, Yang-deficiency constitution; DHC, Damp-heat constitution.
